# Asthenopia and Ocular Headache

**Published:** 1893-06

**Authors:** F. R. Cross

**Affiliations:** Ophthalmic Surgeon to the Bristol Royal Infirmary, and Surgeon to the Bristol Eye Hospital


THE BRISTOL

Medico-Chirurgical Journal.

JUNE, 1893.

### ASTHENOPIA AND OCULAR HEADACHE.

BY
F. R. Cross, F.R.C.S. Eng., M.B. Lond.,
*Ophthalmic Surgeon to the Bristol Royal Infirmary, and Surgeon to the Bristol Eye Hospital.*

The term Asthenopia is applied to eyesight which is easily fatigued, the essential symptom being discomfort in the eyeball, as the result of continuous or even of very moderate eyework. It is most commonly caused by looking at near objects, especially under faulty artificial illumination; but it is very frequently brought on by gazing continuously at a distance, as in watching a cricket match, or at a theatre or picture-gallery. The strain or fatigue often expresses itself outside the eyeball as neuralgia (retro-ocular, supra-orbital, temporal, etc.), and it may cause headache to which the term Ocular Headache has been applied. The headache which is associated with eyestrain is usually located across the forehead, close over the eyebrows. It is frequently one-sided where astigmatism exists in the adjoining eye, and I have then often noticed that it is referred to the anterior portion of the temporal ridge. The pain may be occipital, especially where amblyopia co-exists or there is intra-ocular congestion, but in my experience it is but rarely vertical.

A consideration of this subject is of interest to every practitioner, no less than to the oculist. Any facts which can throw light upon the causation of complaints so common as neuralgia and headache, or which may suggest any plans of treatment likely to help our patients who are suffering from these depressing and obstinate maladies will be a general gain to medical knowledge. On the other hand, it cannot be too widely understood what serious discomforts are being caused by the overpressure of eyes and brain, which seems to be a necessary factor in the education of the present day.

For the purposes of this paper, we may divide ocular neuralgia and headache into two classes:

(1) In which the symptom depends mainly upon some nervous peculiarity of the patient.

(2) In which the cause lies in the eyeball or in its muscular apparatus.

Class I.—It will very rarely occur that the eye and its appendages are absolutely normal, yet a neurasthenic condition of system may be the essential factor in the case, and judicious constitutional treatment the sole means of cure.

In irritable, hysterical, or hypochondriacal patients, especially in those whose eyes are unduly worked—for instance, pupil-teachers, who study for long hours in addition to their duties, bootmakers, seamstresses, and clerks,—symptoms of heaviness and irritability of the eyes, dazzlings under moderate illumination, and failure of sight, are often complained of.

Some of these cases depend on extreme hyperæsthesia of the retina, or of the ophthalmic division of the fifth nerve, the result possibly of former inflammation in the cornea, of which no record may be seen; some, on weakness of the ciliary muscle.

Where there is unstable equilibrium or undue irritability or tendency to exhaustion of the central nervous system, or of the ocular or orbital nerves, symptoms of asthenopia, fatigue, temporary amblyopia, and contraction of the fields may be readily induced; and these symptoms are specially liable to occur under reflex excitation from the nasal and dental branches of the fifth nerve. They have been attributed as reflex phenomena to abnormal conditions of the uterus and ovaries, and even of the intestines.

Conversely in migraine the symptoms are expressed through the fifth and other sensory nerve fibres of the meninges, and through the vagi and sympathetic; but these may be the efferent nerve channels in reflex action, of which the primary afferent impulse starts from irritation of nerve filaments in the eyeball.

In patients liable to headache—the tendency to headache existing even under favourable conditions—it may be induced or exaggerated by eyestrain, a proper correction of which may establish the balance in favour of comfort. Dr. G. M. Gould[1] states that of fifteen hundred cases of headache upwards of seventy-five per cent., and of sick headaches ninety-five per cent., were due to eyestrain. This proportion is largely in excess of my own experience, but no one any longer doubts the extreme importance of careful testing and examination of the eyes in headache.

There is, in my opinion, a typical form of asthenopia, of which I have not seen any account, found in young women who do not show any symptoms of hysteria. The eyes easily tire, and tend to flush and feel hot; there is no impairment of the field of vision, and the refractive error varies, but in many cases is scarcely appreciable, whilst its correction makes but little difference to the symptoms. The essential element is a tendency to active hyperæmia of the face and neck, increased by hot rooms, excitement or study, with, on the other hand, coldness of the hands and feet; in these patients, inquiry must be directed to a better balance of the circulation, and its vaso-motor nerve control, as well as to the condition of the generative and digestive organs.

I think that severe asthenopia is more common in young boys than in girls of the same age, partly perhaps because their studies are more rigorously enforced.

The following case, which has been under my occasional observation for seven years, shows the trouble that may be caused by the idiosyncracy of the patient after slight inflammation:

Basil R., seven years old, came on January 4th, 1886, with slight internal squint of the left eye. This eye was very amblyopic, 20 J.; the sight in it was not sufficient to avoid obstacles in progression. Hypermetropic not more than 1 D.

[1] *Oph. Rev.*, vol. x., p. 280.

The right eye was emmetropic and had perfect sight. After a few months' practice with the left eye, its vision improved to 6/12 1 J.; and later it reached 6/9, where it has remained ever since. He has always seen better without any glasses, even for reading. He was unable to study severely, but went on well, the squint gradually disappearing, until, in May, 1890, he had a slight attack of ophthalmia, which was soon cured; there was no evidence of implication of the cornea, and no follicular swelling remained. From this time, however, any attempt to use his eyes produced lachrymation and headache. His studies were curtailed, but he could not manage them on account of asthenopia. There was no further impairment of the sight, nor could any form of help by spectacles afford relief; he became very excitable, and walked in his sleep. After a short rest in the country he returned apparently well, but broke down again on resuming moderate work. After a full term's rest, he was able to do his lessons in school, his preparation in the evening being done for him. In the beginning of 1892 he completely broke down again—photophobia, lachrymation, and headache,—although his central and peripheral vision remained perfect. Every chance had been given him by his college masters, but I felt obliged to recommend that he should abandon public school life, and be content with a tutor at the seaside. He appeared for two years to have completely recovered, but has recently had a recurrence of his asthenopia independent of any apparent cause.

There is need of a good school where individual attention could be given to boys with asthenopia, progressive myopia, slight zonular cataract, etc., who should not be allowed to attempt the full curriculum of public school studies. For such scholars he aspect and dimensions of the school windows and the method of artificial illumination should be perfect, the seats and desks should be carefully applied, and the amount of reading limited.

Many children come to the hospital complaining of eye-ache and defective vision (5/18 or 5/24), who completely recover with no other treatment than a few weeks' rest; these are cases of ciliary or retinal fatigue, induced by an amount of school work which the children, from some crisis in development or health, are temporarily unable to master. Other types of asthenopia which depend upon some defective conditions in the general health of the patient might be alluded to.

Class II.—The very large majority of the cases of asthenopia depend on faulty conditions in the mechanism of the eye itself, and should be included in a class which comprises errors of refraction and abnormalities of the intrinsic and extrinsic ocular muscles, under the divisions Accommodative and Muscular Asthenopia.

(A) *Accommodative Asthenopia* is the form which is associated particularly with abnormal conditions of the ciliary muscle and with errors of refraction. This division of the subject is very well understood; so much so, that even some medical men seem to consider that an optician's advice as to spectacles is quite sufficient, and fully capable of assisting to cure their patients. Thus encouragement is being constantly given to a form of counter-prescribing which is not creditable to the doctor, nor beneficial to the patient, nor advantageous to the optician, who would still sell his spectacles, if the doctor himself had prescribed them. Those of us who have the greatest experience in dealing with astigmatism or muscular deficiencies know best how difficult it often is to decide what lenses are the most suitable. In an uncomplicated presbyopia there is perhaps no need for a scientific opinion in the selection of glasses. But the reason why a child's sight is deficient or painful, or that of an adult differs from the normal standard in degree or competence, is a problem for solution by medical knowledge.

Donders wrote of asthenopia as a "peculiar morbid condition of the eyes which has long attracted the attention of ophthalmologists. The phenomena of which it is composed are highly characteristic. The eye has a perfectly normal appearence; its movements are undisturbed, the convergence of the visual lines presents no difficulty, the power of vision is usually acute, and nevertheless in reading, writing, and other close work, especially by artificial light, or in a gloomy place, the objects after a short time become indistinct and confused, and a feeling of fatigue and tension come on in, and especially above, the eyes, necessitating a suspension of work."

The emmetropic eye sees at a distance in a state of rest, and its full complement of accommodation is available for closer vision; the hypermetrope, on the other hand, is obliged to tax his accommodation under all conditions where clear vision is required; his focussing muscle is therefore scarcely ever at rest, and when he requires to see more closely only a portion of its normal energy is available. The undue strain may be overcome during youth and health; but as the power of accommodation lessens with age, and particularly under conditions of excessive eyework or of illness or faulty illumination, neuralgia and headache are produced, and the sight fails. It is more than thirty years ago that Donders completely solved the problem of "accommodative asthenopia" and "ciliary strain."[1] His statement that hypermetropia is usually at the bottom of asthenopia remains unchallenged, if we include hypermetropic astigmatism. Still asthenopia may depend upon any condition of refractive error; and this cause is almost universal, for it is only a very small number of eyes that are perfectly emmetropic.

I think that a very large percentage of cases of astigmatism is associated with neuralgia and headache, and that very small degrees of error in curvature of the cornea often give rise to more discomfort than is found in cases where the astigmatic error is more marked. If this be so, it is of prime importance that astigmatic defects should be accurately neutralised, and this can only be done by careful objective testing, as by retinoscopy and the ophthalmometer. No doubt a considerable number of asthenopic patients are wearing with relief spectacles that do not properly neutralise the defect from which they are suffering; on the contrary, there are many others who receive no benefit from partial or inaccurate correction, and in all cases it is necessary to adopt a more scientific method than the subjective one by which the optician aids the patient to select lenses for himself.

Conditions of the ciliary muscle require consideration: presbyopia is the simplest form; fatigue or paralysis of accommodation may occur, notably after diphtheria, in eyes that are otherwise healthy. Insufficiency of the ciliary muscles has been described as a congenital defect;[2] and it may accompany ill-health, at which time asthenopic neuralgia is not infrequently caused by injudicious reading.

Spasm of accommodation is not very uncommon in overworked

1 *On the Anomalies of Accommodation and Refraction of the Eye.* New Syd. Soc. 1864. 2 Theobald, *Trans. Am. Oph. Soc.*

children; by its simulation of shortsight, it is a prolific source of deception in the selection of spectacles by subjective testing. The error caused is almost always spherical. Astigmatism from ciliary spasm I believe to be very rare. For the purposes of a paper on "Retinoscopy,"[1] I examined a large number of eyes by the shadow test and by lenses, both before and after the instillation of atropine; in only a very small number was any change produced in the amount of astigmatic error by the use of the atropine, while the presence of a latent spherical defect was of course frequently shown.

Accommodative asthenopia is caused by an intra-ocular condition which must be sought for in each separate eye; very common in hypermetropia, it may also be present in low degrees of myopia and in myopic astigmatism. The discomforts of shortsight, however, usually depend upon the strain due to inaccurate performance of the act of binocular vision, as controlled by the extrinsic muscles of the eyeball, and constitute one of the forms of (B) *Muscular Asthenopia*.

The best recognised example of this binocular defect is insufficiency of convergence, for in myopia the required amount of convergence is always relatively greater than that of accommodation; while hypermetropia, on the contrary, calls for more accommodation than convergence: and as hypermetropia induces both ciliary failure and ciliary spasm, so in myopia we find insufficiency of the internal recti usually, but sometimes a state of internal strabismus due to continuous overstimulation of these muscles. Treatment consists in the correction of the relative amplitudes of accommodation and convergence by the use of concave spectacles, with, in some cases, the addition of prisms.

Besides the excess of convergence in hypermetropia and the excess of divergence in myopia, which may culminate respectively in internal and external strabismus, there are all sorts of other faulty tendencies in the ocular muscles, causing neuralgias and eye-aches, which are grouped under the term muscular asthenopia.

The defects that cause these symptoms may be present in

[1] *Trans. Intern. Med. Cong.*, 1887.

the muscles or their insertions, in the nerves supplying them, or in the central nervous system. The discomfort depends upon the instinctive adherence to binocular vision, and the undue effort necessary under faulty conditions to maintain it. Thus, as Donders pointed out, if while both eyes of some hypermetropes are fixing an object one be shielded by the hand, the covered eye will rapidly deviate inwards; and conversely, by the same method many myopes, who maintain binocular vision, are shown to have a tendency to divergence, which increases with approximation of the object. In squint and diplopia a faulty position of the eyes is sufficiently obvious; and in cases of monocular amblyopia, where the good eye is alone relied upon for sight, the blurred retinal impression from the other is disregarded, the necessity for binocular vision ceases, and the faulty eye wanders according to its muscular balance.

This tendency to deviation may also exist in eyes with good vision, whether emmetropic or aided by spectacles, but here the effort for binocular vision is usually sufficient to maintain the proper direction of the visual axes, even if muscular asthenopia be thereby induced. If a latent faulty tendency in the muscle balance be suspected, it can be made manifest by rendering one eye amblyopic. For this purpose a strong concave or convex lens, with a stenopæic hole to prevent prismatic action, may be used to blur the image of a lighted candle placed at six metres. A ball of light is seen with one eye, the candle flame with the other; and the relative position of the two as projected by the patient indicates the position of the visual axes. Several methods of reaching the same result have been suggested.

For measuring the statical equilibrium of the eyes, the test must always be made with a distant object—five metres at least,—so that the element of accommodation may be eliminated. For measurements at occupation distance, or for reading, where accommodation and active convergence are called into play, Graefe's test, with the dotted line and vertical prism, has been elaborated by Maddox, who also has given us the glass rod test,[1] so useful in all forms of latent deviation. Moreover, diplopia can always be induced by the use of prisms, and by their means

[1] *Oph. Rev.*, vol. ix., p. 129.

the power of abduction or adduction, or other movements of a pair of eyes, can be approximately tested. The vertical deviation is weak and usually limited to overcoming a prism of 1° or 2°, whilst abduction overcomes 7° or 8°, and adduction is much more powerful.

George Stevens[1] has done much by his work and writings to stimulate our inquiries as to "Anomalies of the Ocular Muscles," and has introduced terms which indicate precisely the special tendency or latent deviation in a given case. In *esophoria* and *exophoria* the eye tends respectively to turn in or out, while if the deviation is up or down *hyperphoria* is present.

I am now in the habit of seeking for latent errors in the extrinsic ocular muscles of many of my patients. Such defects are not very common, but in some cases faulty balance undoubtedly exists, and in such, decentring of the lenses (where ametropia is present) or the use of prisms has relieved the muscular asthenopia.

The value of abducting prisms is well recognised, but I have some half-dozen patients with hyperphoria who wear with comfort vertical prisms. In these cases the essential of treatment lies in orthoptic exercises and the stereoscope. Extravagant statements have been made, particularly by American writers, as to the dependence, not only of asthenopia and headache, but of chorea, epilepsy, and of all kinds of functional nervous diseases upon these ocular defects. It is suggested that cures of the nerve disease in some cases can be brought about by rectifying the defects of equilibrium in the eyes by prismatic lenses, by operations on the recti tendons—graduated tenotomies and partial advancements; on the other hand, among the opponents of these enthusiasts (who, to quote Donders's criticism of Bonnet and Pétrequin—1841,—"are too much preoccupied with the idea of tenotomy") are some who question the entity of muscular asthenopia as distinct from errors of refraction, and entirely deny that ocular derangements can be the cause of nervous disease.

A few cases have been published of epilepsy cured after the use of spectacles. One such case has occurred in my own

[1] New York, 1887.

experience, but I scarcely like to claim the cure as more than a coincidence:

G. R., 21, a very energetic man, 6 feet 3 inches in height, suffered from attacks of petit mal, accompanied by headache, diplopia, and peculiar yellow vision, usually occurring about dinner-time. He complained of slight crossed diplopia, which was corrected by a prism 1° base in, while a prism 3° base in gave him homonymous diplopia.

R. 5/12 J 1 – 1 cyl. \ down and in 5/5
30°
L. 5/6—.50 cyl. down and in ⁄ 5/5
60°

He was fond of reading, and felt no discomfort from it, but he could write for only a short time. He was ordered the cylinders mentioned, to be worn constantly, and happens to have had no fit since.

I made no effort to correct his very weak power of adduction and abduction, which might have been due to hyperphoria (of the existence of which I was at that time ignorant). If spectacles were of value in this case, it was by correction only of the astigmatism.

The following case of faulty muscular balance—mainly hyperphoria—well illustrates this defect and the method of its detection :

C. S., a fine, healthy man of 24, has never been able to use binocularly any race or opera glass. To see through them necessitates the closing of one eye; he never uses them.

For two or three years he has at times noticed that the pictures on his bedroom wall are a little doubled when he wakes in the morning. He has not noticed any discomfort from double vision during the day, but has found that he can sometimes voluntarily get a double image. For some months, when tired, he has had slight difficulty in reading; the lines tend to become doubled one above another, and unsteady: this is at once relieved by closing either eye, and his friends have noticed that he has got in the habit of putting his hand involuntarily to his left eye when he is tired in reading.

Vision : R. 6/6 emmetropic
L. 6/8 + .50 cyl. 6/6.

Has been given + .50 readers, which are no help. Asthenopia is at times definite, but never severe.

Testing him with a candle at six metres, with a red glass over one eye, no diplopia could be elicited; but single vision was upset by a prism 9° apex in, or 4° apex out, showing marked weakness both of adduction and abduction. Whereas the movements of the right eye upward, and of the left down, were unusually powerful, single vision being maintained with a prism 5° apex up on the right and 4° down on the left, and a prism of 3° in the same positions gave a feeling of relief to the strain felt when the eyes were unaided; on the other hand, double vision was at once produced by a weak prism 1° apex down over the right, or apex up over the left.

The patient looking at a candle flame at six metres' distance with a strong lens and stenopæic opening over the right eye, the blurred image seen was below and to the right of the candle seen naturally with the left eye.

Maddox's rod placed horizontally over the right eye, gives that eye the impression of a vertical line of light (in orthophoria the light line and candle should overlap). In C.S. the streak wandered about 30 cm. to the right, showing that the eye was turning inwards (esophoria). A 5° prism, apex in, corrected the abnormality. When the glass rod is placed vertically, it gives the impression of a horizontal bar of light; thus placed over the right eye, the bar of light deviated to the base of the candle-stick, showing that the eye had wandered upwards; placing the bar over the left eye, the converse obtained—the bar was projected upwards by passive deviation of the eyeball downwards.

The faulty tendency was rather more marked when the right eye was rendered amblyopic than the left; so that while a prism apex down 6° corrected the left, a prism apex up of 8° was required to correct the right.

Although no diplopia could be made manifest without the use of prisms, or by some method by which the vision of one of the eyes was rendered indistinct, the patient at once derived great comfort from placing a 3° or 4° prism apex up over the right eye, and with the same lens read quite comfortably.

The asthenopia has been corrected by spectacles:

R. Prism 2° apex up

L. + .50 cyl.

A prism of 4° was to be used vertically wrong ways, so as to exaggerate the diplopia and thus stimulate the faulty muscles, and the stereoscope was prescribed for orthoptic exercise.

A recent paper by Dr. H. F. Hansell,[1] on "The Prominent Symptoms of Hyperphoria, as illustrated by thirteen successive cases," indicates the importance of enquiring into the conditions of the muscular equilibrium of the eyeballs. In the cases described all causes of discomfort, at any rate all ocular defects, excepting the muscular anomaly named, are said to have been excluded. The symptoms complained of by the patients were asthenopia, lachrymation, photophobia, headache, in some cases

[1] *Med. News*, Feb. 18, 1893.

attended by vomiting, vertigo, dizziness, dyspepsia, nervousness, irritability of temper, mental confusion. These varied types of abnormal conditions are said to have been relieved by curing the hyperphoria, either by tenotomy or by the use of prisms.

